# Comparative experimental re-evaluation of the two implanting methods of silicone gel testicular prostheses in beagle dogs

**DOI:** 10.1186/1756-0500-4-99

**Published:** 2011-04-02

**Authors:** Tong-li Hao, Peng Chen

**Affiliations:** 1 Department of Urology, General Hospital of Chinese People's Liberation Army, Beijing, China; 2 Department of Electromagnetics and Laser Biology, Radiation Medicine Institute, Academy of Military Medical Sciences, Beijing, China

## Abstract

**Background:**

Testicular prosthesis has been applied clinically for decades, and implantation of testis prosthesis under the tunica albuginea has been considered to be the standard method in the most of the reports. However, postoperative scrotal appearance, the mobilization and the palpitation of the prosthesis are not always satisfactory to all the patients. Modifications in surgical techniques might be necessary to bring improvements to the clinical outcomes in testicular prosthesis implantation.

**Findings:**

In a group of 9 beagle dogs in this study, an orchiectomy succeeded with a testicular prosthesis implantation under the tunica vaginalis, and a complete mechanical denudation of the testicular parenchyma succeeded with an implantation under the tunica albuginea were performed, respectively. Histopathological evaluations of the scrotal tissues and the implants, which were made at the end of the follow-up, showed that all the tested animals lived uneventful lives during the follow-up period, and no rejections or infections were found. Prostheses implanted under the tunica vaginalis showed a more satisfying mobilization and palpation than those implanted under the tunica albuginea. Chronic inflammation in the para-prosthesis tissues with vascular proliferation and fibrinogenesis were more common in the "under tunica albuginea" group than that in the "under tunica vaginalis" group, although differences in fibrinogenesis between the two groups were found to be statistically insignificant.

**Conclusions:**

In this comparative study, we have re-evaluated the two most popular implantation methods of testicular prosthesis, the "under the tunica albuginea" and the "under the tunica vaginalis" pathways, in animal models. We found that the testicular prosthesis were all well tolerated, but the prosthesis implanted under the tunica vaginalis showed a more satisfying result concerning appearance, palpability, and histopathological findings than that of the "under the tunica albuginea" group. The "under the tunica vaginalis" method might become a more practical method for future testicular prosthesis implantation.

## Background

The psychological effect of castration secondary to the loss of testes as a result of testicular trauma, cancer or spermatic cord torsion often results in negative impacts to the lives of the patients. In the past, prostate cancer was once considered to be rare in the Chinese population. Nowadays, the incidence of prostate cancer in the Chinese population is increasing rapidly. As an alternative to surgical treatment of prostate cancer, orchiectomy is often performed. In China, during the past several decades, fewer demand for reconstruction of the "vacant" scrotum was actively requested by these patients. With the social and economic changes in China, the demand for testicular prosthesis is increasing rapidly now. However, postoperative pain still remains a relatively common problem for the testicular prosthesis recipients. The relationship between the surgical technique and the location of the prosthesis remains unclear. In the present study, comparative observations concerning the effects of silicone gel testicular prostheses implanted in different locations, "under the tunica albuginea" or "under the tunica vaginalis", in Beagle dogs are reported.

## Methods

The testicular prostheses used in the present study were a product of the Weining Rubber Product Company (Shanghai, China). The prostheses consisted of two components, silica gel as the filler, and silicon rubber as the shell. According to pre-operative measurements of the testicles, the prostheses were manufactured in the range of 4.0 to 6.0 ml, and sterilized with ethylene oxide prior to use. The safety and biocompatibility of the prosthesis has been tested in accordance with the institutional review board.

Overall, nine healthy adult male Beagle dogs were enrolled in the present study. The study protocol was approved by the Institutional Ethical Committee.

Levels of serum testosterone, kidney function, and liver function of all the dogs were tested before and after the implantation of testicular prosthesis.

Following intravenous administration of a mixture of Sumianxin II (Institute of Animal Science, Changchun University of Agriculture and Animal Sciences) and ketamine (Shanghai No.1 Biochemical Pharmaceutical Co., Ltd.) in a 1:1 ratio, a midline scrotal incision was made in each side of the scrotum. For the left, a longitudinal incision in the tunica albuginea was made, and the testicular parenchyma was carefully denuded bluntly with a stainless steel scalpel holder. For homeostasis, the inner surface of the tunica albuginea was gently pressed with gauze. For the right, a routine orchiectomy was performed with the spermatic stump transfixed using silk suture.

**Figure 1:**
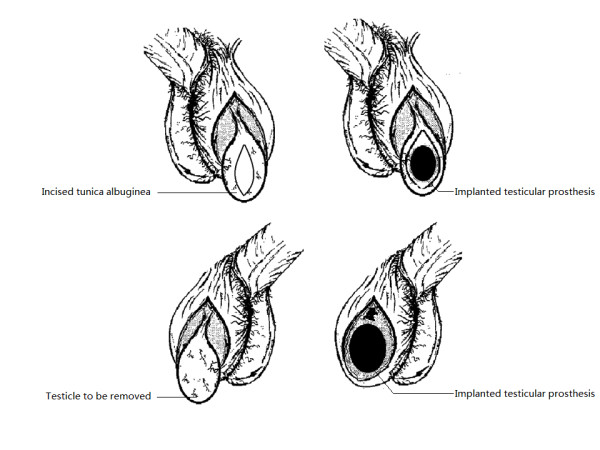
**Illustration of surgical procedures regarding the implantation of the testicular prosthesis**. For the left scrotum, the testicular prosthesis was placed under the tunica albuginea, and for the right, under the tunica vaginalis.

A testicular prosthesis of appropriate size was implanted in different ways. For the left, the prosthesis was placed under the tunica albuginea, and the tunica albuginea was closed with an absorbable suture. For the right, the prosthesis was placed under the tunica vaginalis. After these procedures, the scrotum was closed in multiple non-overlapping layers with absorbable sutures. No drains were left in the surgical sites (Figure 1).

NSAIDS (Indomethacin Suppositories, 25 mg) was given to the dogs every 12 hours in the first 7 postoperative days. The animals were followed up for a period of 3 months, and the testis together with the whole scrotum was removed en bloc at the end of the observation. All the samples were preserved in 4% formaldehyde solution for later histopathological evaluations.

Qualitative and quantitative evaluation under a standard light microscope for levels of chronic inflammation and fibrosis were made according to the criteria below referring to the Karademir's Method with appropriate modifications [1]:

### Chronic Inflammation

The presence of plasmocytes, hystiocytes, lymphocytes as well as vascular proliferation in the tissues around the prosthesis or testicle was graded from 0 to 3.

0 = No inflammation.

1 = Focal inflammation areas with little vascular proliferation.

2 = Medium level inflammation, where sparse inflammation exists in all microscope fields.

3 = Significant inflammation with dense infiltration and vascular proliferation.

### Fibrosis

The fibrohyalinization in the surrounding tissue was evaluated. All the parameters were measured in 10 separate microscopic fields from different sites.

Grade I: Focal and mild fibroblastic activation.

Grade II: Diffuse mild fibroblastic activation.

Grade III: Significant fibroblastic activation.

For statistical evaluation, SPSS 16.0 software was used. Variables are presented as mean ± SD. A Mann-Whitney U-test was utilized for data analysis and a level of p < 0.05 was considered to be statistically significant.

## Results

During the follow-up of 3 months, all the nine research subjects survived uneventfully with no apparent weight loss. Postoperative slight scrotal edema was found in both side of the scrotum and lasted for 3 days and 7 days for the right and the left, respectively. Rejection and infection were not found in any of the prosthesis-implanted dogs. In all the dogs, the scrotal wounds healed within a week after surgery, and proliferative scar or keloid tissue were not present. The testicular prostheses were mobile and palpated at normal localizations in the right scrotum. However, in 7 out of the 9 dogs, prostheses in the left scrotum were found to be slightly asymmetric to and hanged higher than that in the right. As for the palpation, the prosthesis in the left scrotum seemed to be more rigid than that in the right.

The prostheses were removed en bloc together with the scrotal wall at the end of the follow- up. All the prostheses were well enclosed by surrounding tissues, and no apparent interspace was found between the tunica vaginalis and the tunica albuginea in the left scrotum. No macroscopic leakage of the contents from the testicular prostheses was identified. The prostheses could be easily detached from the tunica albuginea or the tunica vaginalis.

**Figure 2:**
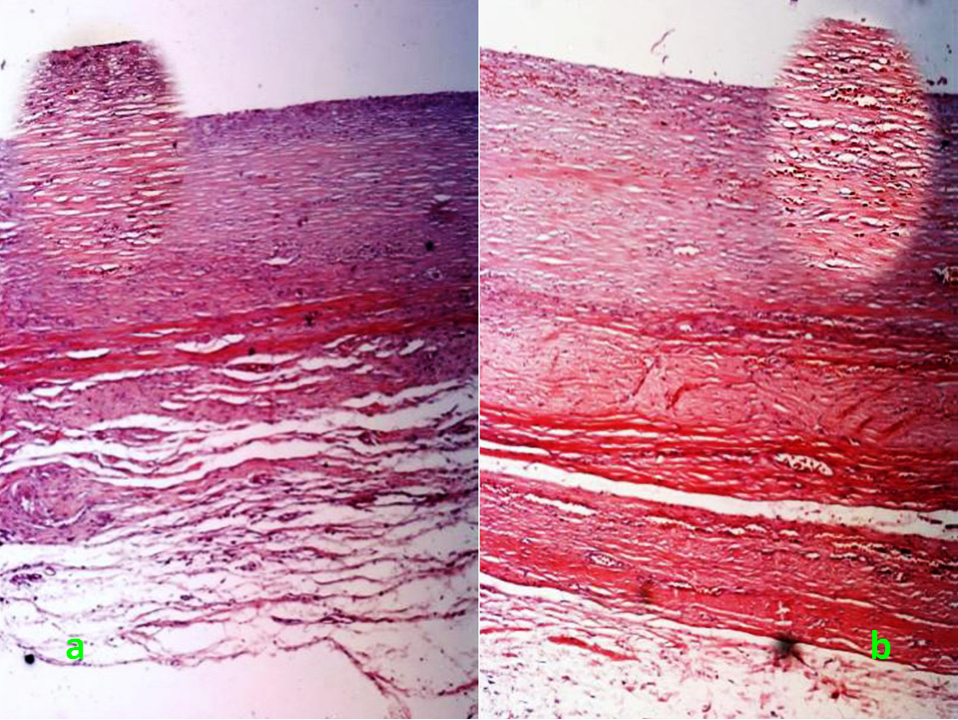
**Chronic inflammation and fibrogenesis in the pseudo capsule of the testicular prosthesis**. Magnification ×100 for each image, and ×200 for each corresponding oval inserted image.

Histopathological evaluations revealed that chronic inflammation and vascular proliferation were present in both sides of the scrotum. These findings in the tunica albuginea seemed to be more apparent in the left (Figure 2a), in contrast to that in the right (Figure 2b). The differences between the two sides were found to be significant with respect to chronic inflammation, though it was insignificant as it comes to fibrosis. Fibrogenesis in the surrounding tissues could also be found in both of the two sides (Table 1 and Table 2).

**Table 1:** Comparison of chronic inflammation between the two groups

	Left (n = 9)	Right (n = 9)	*p *Value	Z
**No inflammation**	0	3 (33.33%)	0.011	-2.749
		
**Mild**	5 (55.56%)	5 (55.56%)		
		
**Moderate**	4 (44.44%)	1 (11.11%)		
		
**Severe**	0	0		

**Table 2:** Comparison of fibrosis between the two groups

	Left (n = 9)	Right (n = 9)	*p *Value	Z
**No fibrosis**	0	0	0.436	-0.922
		
**Mild**	3 (33.33%)	5 (55.56%)		
		
**Moderate**	6 (66.67%)	4 (44.44%)		
		
**Severe**	0	0		

Before the surgery, the average serum testosterone of the dogs was 11.05 ± 1.85 nmol/L. One week after implantation of the testicular prosthesis, it decreased to a level of undetectable to the Radioimmunoassay (RIA) method. No significant differences with respect to liver and kidney function were found between each paired group during the follow-up (Table 3).

**Table 3:** Liver and kidney function before and after implantation of testicular prosthesis ( ± s, n = 9)

**Index**	**Before**	**After**			
		
		**7**	**30**	**60**	**90**
		
ALT(U/L)	36.2 ± 5.3	39.2 ± 4.3	37.5 ± 5.1	32.3 ± 6.6	30.8 ± 5.4
AST(U/L)	51.1 ± 6.3	49.6 ± 5.1	48.8 ± 6.1	50.5 ± 4.7	46.3 ± 3.8
γ-GT(U/L)	40.5 ± 3.6	38.1 ± 2.7	40.2 ± 3.8	39.7 ± 5.5	37.2 ± 4.6
T-BIL(μmol/L)	3.5 ± 0.6	3.6 ± 0.3	2.5 ± 0.3	4.0 ± 0.5	3.1 ± 0.2
ALB(g/L)	37.0 ± 8.1	38.5 ± 7.6	32.8 ± 6.1	35.5 ± 5.3	30.1 ± 4.9
Cr(μmol/L)	66.5 ± 3.9	65.4 ± 4.2	57.5 ± 3.0	60.6 ± 4.2	58.3 ± 6.1
BUN(mmol/L)	2.3 ± 0.1	2.6 ± 0.3	3.1 ± 0.2	2.9 ± 0.3	2.4 ± 0.3
UA(μmol/L)	27.6 ± 3.1	29.1 ± 2.8	30.2 ± 2.2	27.9 ± 1.8	30.5 ± 2.2

ANOVA Analysis, differences between each paired group was not statistically significant, p > 0.05.

## Discussion

Testicular prosthetic devices have been developed to restore the normal appearance of the scrotum and hopefully to restore quality of life. The first testicular prosthetic device was introduced in 1939 by Bowers using the metal alloy vitalium [2]. The practicality and safety of testicular prostheses has been tested clinically for several decades, especially following the immergence of the silicone gel prosthesis in the 1970s [3]. Despite the concerns over the relationship of silicone implants to connective tissue disease [4-6], multispecialty expert panels in the United States (Institute of Medicine and the National Science Panel) and the United Kingdom failed to find evidence which could indicate any causal linkage between them. Silicone gel filled implants are currently still widely used all over the world, especially in China [7].

For successful testicular prosthesis implantation, the key to the surgical procedure is the correct positioning of the prosthesis in right location within the scrotum. Surgically, the traditional inguinal procedure for testicular prosthesis implantation has been applied for a long time, and has proven to be minimally successful in maintaining the mobility and sensibility of the "testis". For this traditional method, there is a possibility of inguinal migration of the prosthesis, spontaneous exit of the implant, infection, hemorrhage, breakage of the implant, or persistent pains. With developments in surgical techniques, testicular prosthesis implantation techniques have improved greatly, and scrotal procedures with the testicular prosthesis implanted under the tunica albuginea after denudation of the testicular parenchyma has been practiced for many years. According to the literature, this technique has proven to be successful, especially with regard to cosmetic appearance (mobility and sensibility, etc.) [8]. Despite these benefits, clinical observations have indicated that a postoperative pain rate in 1% to 5%, or even to 9% can be found in the prosthesis recipients [9-11]. In the past, the majority of the recipients of testicular prosthesis in China were those with unilateral or bilateral testicle loss from scrotum injuries, testicular cancers, or testicular torsions. Nowadays, with the increasing incidence of prostate cancer, the demand for testicular prosthesis implantation has also increased rapidly. In our clinical practices, scrotal (under the tunica vaginalis) or subalbugenous (under the tunica albuginea) implantation of the testicular prosthesis are the two most popular surgical methods performed in this field. Despite a relative higher general satisfaction rate to these procedures in most of the recipients, discomforts, especially postoperative pains, have become a common problem. This phenomenon exposed the need for a comparative re-evaluation between these two methods on a basis of scientific study. For a successful testis prosthesis implantation, the recipient's satisfaction is a very important issue. However, it is a subjective problem, and we could only get it from human recipients. As for an intensive investigation, especially the histopathological study, we have to use animal models. In fact, human recipients' satisfaction with their testis prosthesis had been reported in a lot of literatures in the past. Because the focus of this study is on those objective issues, such as the reasons for pains, asymmetric appearances, and palpations, etc., so we decided to take an animal model.

In this perspective controlled animal study, a popular testicular prosthesis material, silicone gel, is used to obviate the interference from prosthesis materials. In order to maintain a relatively reasonable comparability, testicular prosthesis are implanted in each side of the scrotum but at different locations, with one inside the scrotal cavity (under the tunica vaginalis) after regular orchiectomy and another under the tunica albuginea following denudation of the testicular parenchyma. During a follow-up of 3 months, we found no incidence of infection, rejection or rupture of the implants, and all the 9 dogs survived uneventfully. These results indicate the safety of the silicone gel prosthesis utilized. Apparently, this is not a indicative of the superiority of this testicular prosthesis to the materials used in other studies [12].

In animal experiments, one can not objectively quantify the subjective feelings of recipient animals. However, postoperative scrotal appearances and histopathological findings might provide useful information. In the present study, we found that in 7 out of the 9 animals, the bilateral scrotum were not symmetric, with the left "testicle" in a relative higher position. On the one hand, we attribute this to the equal gravity but unequal support to the prosthesis in the bilateral scrotum, where there is the spermatic cord acts as a support for the left "testicle", but not for the right. On the other hand, we speculate that the spontaneous contraction of smooth muscle in the spermatic cord in the left might also play an important role. All these facts naturally lead us to contemplate some deep initiative factors in behind. Histopathological findings in our study revealed that slight to moderate inflammation and vascular proliferation in the left scrotum are more common than that in the right throughout the entire follow-up period of 3 months. According to these findings, we could naturally speculate that chronic inflammation might act as a very important stimulating factor for the contraction of the spermatic smooth muscles and thus the subsequent elevation of the left "testicle". It could also be speculated that this might be a result of the denudation of the testicular parenchyma, which could lead to edema and subsequent adhesion of the tunica albuginea to the surrounding tissues.

In contrast to the left scrotum, chronic inflammation and fibrosis are relatively slight in the right. Except for the lack of the denudation procedure of the testicular parenchyma, the good elasticity and abundant blood supplies of the scrotal tissues might help to alleviate the inflammation and provide a suitable accommodative environment for the testicular prosthesis. All these findings imply that the presence of chronic inflammation might even be the very cause of postoperative pains in the prosthesis recipients. Recently, some researchers have reported that silicone could be used as inducing materials in peritoneal cavity to produce a kind of mesothelium-lined fibroblast-rich tissue for tissue-engineering purposes. These findings are indicative of a better compatibility of silicone materials in peritoneal or scrotal tissues. In this study, we have also found a thinner layer of mesothelium-lined fibroblast-rich tissues in the inner surface of the false capsule in the right side [13].

## Conclusions

In this comparative study, we have re-evaluated the two most popular implantation methods of testicular prosthesis, the "under the tunica albuginea" and the "under the tunica vaginalis" pathways, in animal models. From this study, we found that the testicular prosthesis were all well tolerated, but the prosthesis implanted under the tunica vaginalis showed a more satisfying result concerning with appearance, palpability, and histopathological findings than that of the under the tunica albuginea group. The "under the tunica vaginalis" method might be a more practical method for future testicular prosthesis implantation.

## Competing interests

The authors declare that they have no competing interests.

## Authors' contributions

TLH and PC contributed equally to this manuscript. They designed, planned and performed all the experiments, including surgical operations on the animals, the evaluation of the pathological findings, and the writing of this manuscript. They all participated in data analysis and figure generation, interpretation and writing of the manuscript. All authors have read and approved the final version of the manuscript.
